# Royal Somerset Jennerian Society

**Published:** 1806-04

**Authors:** 


One of the Surgeons of the Royal Somerset Jennerian Society.

---

### *Royal Somerset Jennerian Society.*

THIS Society have much pleasure in reporting to the public, the result of their endeavours for the promotion of Vaccine Inoculation during the past year; and although it will appear, that from the opposition of prejudice, interest and ignorance, the numbers of poor vaccinated in the city of Bath are but comparatively small, yet the Society have well-grounded hopes, from their general success, that by steady perseverance on their part, every difficulty will (in a very few years) be overcome, and the small-pox, once considered as a plague, will no longer exist.

It is with the utmost satisfaction they have to state, that even in the first year of this institution, no less than 1429 persons have received from it the benefits of Vaccine Inoculation; of these, 1075 were vaccinated at the twenty-eight stations appointed by them in the county of Somerset for gratuitous inoculation, and only 354 at the principal station in Bath; and it is with the deepest regret the Society reflect on the number of persons who have suffered affliction, disease and death, from the natural small-pox during the last twelve months in this city, when so safe and and certain a preventive was gratuitously offered to them by this Society.

In the number vaccinated by this Society either in Bath or the county of Somerset, not a single instance has occurred of death, of indisposition (but of the slightest kind), or of subsequent disease.

The Society have also additional and well-founded satisfaction in observing, that although the casual small-pox has raged with unusual virulence during the late summer, and though the vaccinated persons have been frequently exposed to its most active contagion, yet in *no one instance*, which has come to the knowledge of the Society, has small-pox succeeded to vaccination. Supposed cases have, indeed, been brought forward in the public papers; but these, on fair, candid, and patient investigation, have been proved to be totally unfounded.

Their principal station has afforded a constant supply of perfect and genuine vaccine virus to the medical gentlemen and others in Bath and the county of Somerset.

Instructions for the due and efficient practice of Vaccine Inoculation, together with plans of the Institution, have been circulated throughout the county (a measure the Society have every reason to believe essentially important), and every possible publicity has been given to the undertaking by advertisements in the public papers, handbills, &c.

In the prosecution and extension of their important designs, the Society hope for the continuance of public support, and flatter themselves they have a more than common claim on their liberality, both with respect to pecuniary aid and general concurrence; as there is hardly a physical evil, the abolition of which would be more beneficial to society than that of the small-pox.

The Society have the honour of acknowledging the great approbation of their proceedings, expressed by the Magistrates assembled at the quarter-sessions of the peace of the county of Somerset.

The Surgeons of the Royal Somerset Jennerian Society attend at the Bath City Dispensary every Monday, at three o'clock, for the gratuitous vaccination of those who apply.

Subscriptions received at Messrs. Hobhouse and Co's Bank, Bath.

